# Cold agglutinin syndrome as a precursor for the diagnosis of low‑grade lymphoma: A case report

**DOI:** 10.3892/mi.2024.211

**Published:** 2024-12-18

**Authors:** Hüseyin Döngelli, Güner H. Özsan

**Affiliations:** 1 Department of Internal Medicine, Dokuz Eylul University Hospital, Izmir 35000, Turkey; 2 Department of Hematology, Dokuz Eylul University Hospital, Izmir 35000, Turkey

**Keywords:** cold agglutinin syndrome, hemolytic anemia, lymphoma, peripheral blood smear, renal cell carcinoma

## Abstract

Cold agglutinin syndrome is a form of acquired hemolytic anemia that typically arises from underlying conditions, such as infections, autoimmune disorders or lymphoid malignancies. The majority of patients remain asymptomatic and are diagnosed with anemia through routine complete blood count (CBC) testing. The present study describes the case of a male patient in his 50s who sought a second opinion at the authors' clinic due to newly detected anemia. A peripheral blood smear revealed red blood cell (RBC) agglutination, and testing confirmed the presence of cold agglutinin antibodies. Thoracoabdominal computed tomography identified a mass in the right kidney, leading to a partial nephrectomy. A pathological examination of the mass confirmed a diagnosis of renal cell carcinoma. At that time, no secondary cause for the cold agglutinin syndrome was identified. At 2 years following his initial admission, the patient presented with bilaterally enlarging masses in the groin. A biopsy was performed, which revealed low-grade B-cell lymphoma. Following R-CHOP treatment, the RBC agglutination was resolved, and the CBC returned to normal ranges. On the whole, the present study demonstrates that cold agglutinin syndrome may serve as an early indicator of lymphoma.

## Introduction

Cold agglutinin disease (CAD) is a rare form of cold autoimmune hemolytic anemia. Also referred to as primary CAD, this chronic condition is characterized by complement-mediated hemolysis and non-progressive, clinically non-malignant B cell proliferation. CAD should be considered in the differential diagnosis of patients presenting with unexplained anemia, with or without associated cold-related symptoms, such as acrocyanosis, livedo reticularis, Raynaud's phenomenon and fingertip ulceration. Notably, nearly all cold agglutinins test positive for the C3d direct antiglobulin test (1).

Cold autoimmune hemolytic anemia (AIHA) can be classified into primary CAD and secondary cold agglutinin syndrome (CAS), with the latter often triggered by underlying conditions, such as infections (e.g., mycoplasma pneumoniae, Epstein-Barr virus or influenza A), malignancies (e.g., diffuse large B-cell lymphoma, Hodgkin lymphoma or metastatic melanoma) and autoimmune diseases (e.g., systemic lupus erythematosus). In the differential diagnosis of cold AIHA, other causes of hemolysis should also be considered, including warm AIHA, hereditary hemolytic anemias (e.g., hereditary spherocytosis or G6PD deficiency), paroxysmal cold hemoglobinuria and drug-induced hemolysis. Cold-induced hemolysis can also occur in cases of hypothermia or mechanical damage to red blood cells (RBCs), although these do not typically result in cold agglutination. The diagnosis of cold AIHA involves clinical evaluation, testing for cold agglutinins, direct antiglobulin tests and the careful exclusion of other potential causes of hemolysis, such as infections, malignancies, or hereditary conditions (2).

For the majority of patients without severe symptoms, a strategy of watchful waiting is appropriate. Conventional treatment options, such as corticosteroids, azathioprine, or cyclophosphamide which are typically effective in warm antibody-mediated AIHA, are ineffective for CAD. Moreover, a splenectomy is not beneficial in CAD, as hemolysis predominantly occurs in the liver. Rituximab monotherapy is considered the first-line treatment for CAD. Other treatment options include the use of bendamustine, combinations of rituximab and bendamustine, ibrutinib, acalabrutinib, venetoclax, eculizumab and sutimlimab (3,4). Currently, to the best of our knowledge, there is no specific treatment option available for secondary CAS other than addressing the underlying disease.

The present study describes the case of a male patient who sought a second opinion at the authors' clinic due to newly detected anemia.

## Case report

A 53-year-old male patient presented to the Department of Hematology, Dokuz Eylul University Hospital, Izmir, Turkey, seeking a second opinion regarding anemia newly detected during routine check-ups. The patient, who reported no symptoms, had a medical history of diabetes, hypertension and inactive chronic hepatitis B virus (HBV) infection for the past 10 years. He was being treated with metformin, losartan and amlodipine. His family history did not reveal any significant findings. The patient had been a smoker for >20 years and consumed alcohol socially.

A physical examination revealed no abnormalities. Prior to his visit to the Department of Hematology, Dokuz Eylul University Hospital, two separate complete blood count (CBC) analyses were conducted at two different hospitals the previous week, yielding conflicting results: One report indicated normal findings, while the other indicated macrocytic anemia.

A CBC, hemolytic parameters (including lactate dehydrogenase, bilirubin, haptoglobin and reticulocyte count), and a peripheral blood smear were performed. Routine blood tests, including liver and kidney function tests, yielded results which were within normal range. The initial CBC results indicated an elevated mean corpuscular volume and mean corpuscular hemoglobin concentration, alongside a decreased RBC count and hematocrit, while hemoglobin levels remained within the normal range (Table I). The peripheral blood smear revealed agglutination of the RBCs. Other tests, including hemolytic parameters, yielded results which were within normal limits.

Further analyses revealed negative cryoglobulin and mycoplasma antibody test results, normal blood immunoelectrophoresis, a positive direct Coombs test (C3d), and a positive cold agglutinin titer of 1:256 at 4˚C, leading to a definitive diagnosis of cold AIHA. A second CBC and peripheral blood smear conducted with warmed blood samples yielded normal CBC results (Table I) and the disappearance of RBC agglutination.

**Figure 1:**
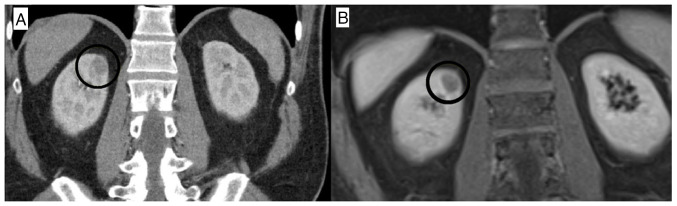
(A) Abdominal computed tomography scan indicating a mass of 2 cm in size (black circle); (B) magnetic resonance imaging illustrating a mass consistent with renal cell carcinoma (black circles indicate renal cell carcinoma).

A whole-body computed tomography (CT) scan revealed a mass 2 cm in size in the right kidney (Fig. 1A). Magnetic resonance imaging of the mass suggested renal cell carcinoma (RCC) (Fig. 1B). Subsequently, the patient underwent partial nephrectomy, and a pathological examination confirmed the diagnosis of RCC. No other abnormalities were noted on cross-sectional imaging. Notably, no secondary cause of cold AIHA was identified. A bone marrow biopsy was performed, revealing no malignancy or evidence of cold agglutinin-associated lymphoproliferative bone marrow disease.

The patient was routinely monitored, and the CBC remained consistent with the initial findings. The persistent agglutination of RBCs was noted in the peripheral blood smear, with no notable hemolysis observed during follow-up.

**Figure 2:**
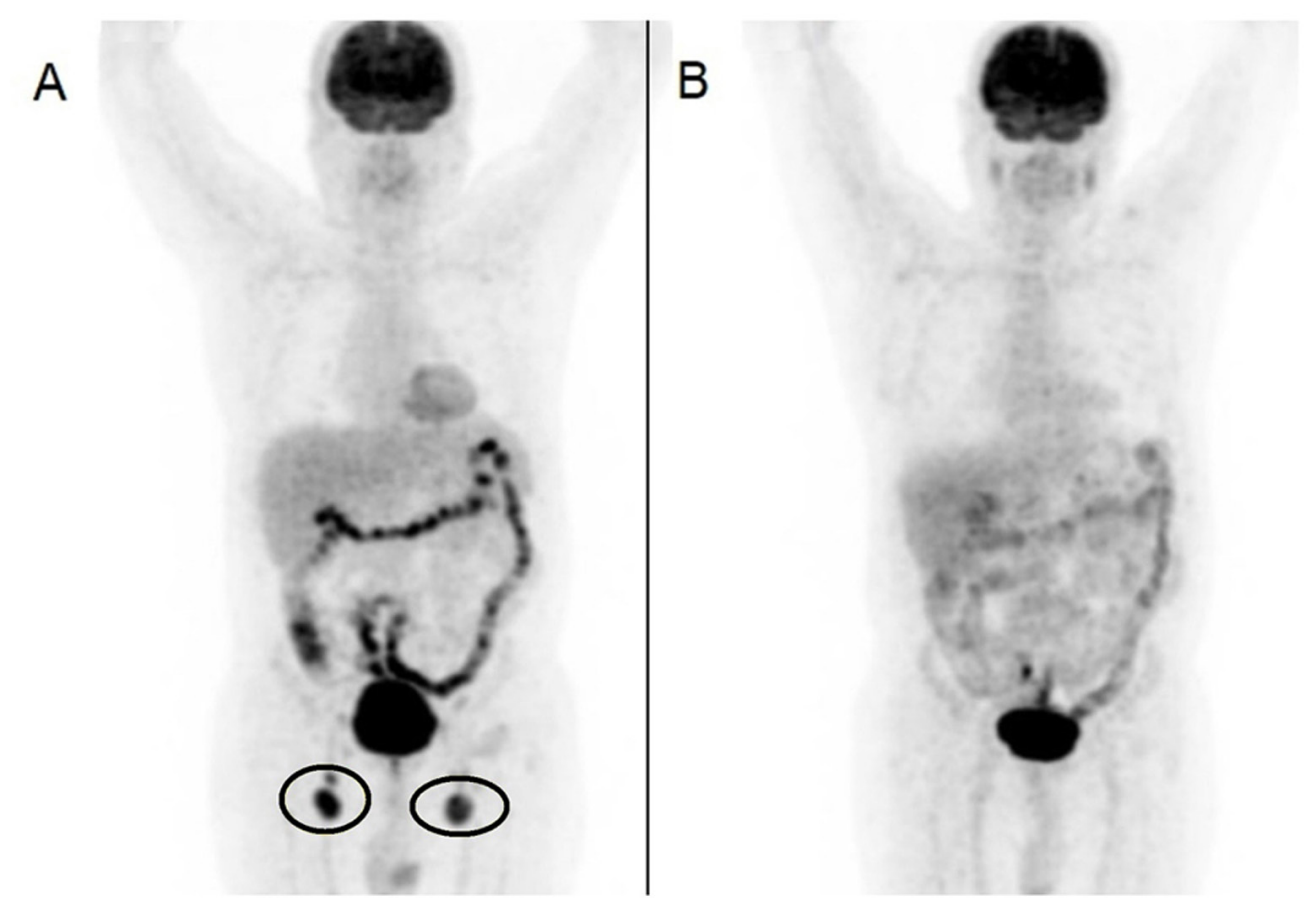
FDG PET/CT scans, performed (A) before and (B) after R-CHOP treatment, taken 3 months apart, illustrating the metabolic response of the lymphoma, with a decrease in the previously diffuse increase in FDG uptake in the inguinal lymph nodes (indicated by black circles). The diffuse FDG uptake in the intestine observed on (A) the first PET/CT scan was attributed to metformin use. FDG, fludeoxyglucose-18; PET, positron emission tomography; CT, computed tomography.

At 2 years following his initial visit, the patient presented to the hematology department with complaints of a growing mass in the groin. He did not report any symptoms of B-cell lymphoma, such as unexplained weight loss, night sweats, or fever. The initial examination revealed hepatosplenomegaly and a bilateral enlargement of the inguinal lymph nodes. A CBC indicated lymphopenia and marked anemia. Similar to the first presentation, the agglutination of erythrocytes was observed in the peripheral blood smear. A fludeoxyglucose-18 (FDG) positron emission tomography (PET)CT scan was requested, and the results were consistent with lymphoma (Fig. 2A). A biopsy was performed on the right inguinal lymph node, and histological examination revealed low-grade B-cell lymphoma. A repeat bone marrow biopsy was conducted, and histological analysis reported hypercellular bone marrow without evidence of clonal disease, lymphoma infiltration, or cold agglutinin-associated lymphoproliferative bone marrow disease.

Routine laboratory examinations prior to initiating chemotherapy did not detect any abnormal findings, including aspartate aminotransferase, alanine aminotransferase, kidney function tests and liver function tests. Moreover, it is standard procedure in Turkey to screen for HBV infection prior to chemotherapy: HBsAg was positive as was expected, and HBV-DNA was positive, although at an undetectable level. Therefore, tenofovir alafenamide was initiated as HBV reactivation prophylaxis. No HBV reactivation was detected before or after chemotherapy.

The patient was subsequently initiated on R-CHOP therapy (cycle repeats every 21 days), consisting of rituximab (375 mg/m^2^ intravenously on day 1), cyclophosphamide (750 mg/m^2^ intravenously on day 1), doxorubicin (50 mg/m^2^ intravenously on day 1), vincristine (1.4 mg/m^2^ intravenously on day 1) and prednisone (40 mg/m^2^ perorally on every day of first week in every cycle). A second FDG PET/CT was ordered, and the patient exhibited a complete response after two cycles of R-CHOP treatment (Fig. 2B). It was decided to complete a total of four cycles of R-CHOP. Following this regimen, the patient began rituximab maintenance therapy (375 mg/m^2^ intravenously every 6 months). Peripheral blood smears were performed after the second and fourth cycles of chemotherapy, as well as at the 6-month mark following the completion of chemotherapy. Post-treatment, the CBC and peripheral blood smear returned to normal limits, and the erythrocyte agglutinations previously observed in the peripheral blood smear conducted with unheated blood were no longer present.

## Discussion

In the case described in the present study, there was no true anemia or associated symptoms at the initial presentation; however, anemia is the most prevalent finding in cold AIHA, occurring in 51% of patients at diagnosis (5). The first indication of CAS was the discrepancy observed between two CBCs performed prior to the admission of the patients to hospital. Subsequent re-examinations using heated blood samples revealed the disappearance of erythrocyte agglutination in the peripheral blood smear, alongside a return to normal CBC values, which were crucial for the diagnosis of CAS in this instance. The findings on the CBC were contradictory, as the degree of RBC agglutination is dependent on the environmental temperature and the duration of exposure. Various factors, including the time from obtaining the blood sample to receiving the laboratory results, could affect the impact of environmental temperature on the degree of agglutination.

Terán Brage *et al* (6) presented an intriguing case of CAS in a patient with renal carcinoma; a male in his 60s was diagnosed with paraneoplastic CAS, while being evaluated for microcytic anemia with evident hemolysis. In the case described herein, RCC was incidentally identified during the investigation of secondary causes, and while the tumor was excised, the findings of CAS persisted, suggesting no clear association between RCC and CAS in this instance. Notably, the patient in the present study exhibited no signs of anemia or hemolysis, and the initial CBC erroneously indicated macrocytic anemia due to erythrocyte agglutination. Furthermore, cold AIHA may also manifest in renal findings; Taberner *et al* (7) reported a case of renal hemosiderosis associated with CAD.

Additionally, there are case reports linking CAS to lymphoma (8,9). In the present case report, CAS was identified during the evaluation of conflicting CBC results; the patient remained asymptomatic, with no true anemia or clear hemolytic signs. While other case reports have diagnosed lymphoma during the investigation of CAS, the patient in the present study was diagnosed with lymphoma 2 years after CAS was detected (8-10).

CAS may serve as an early indicator of lymphoma, as supported by previous case reports in the literature (10,11). Following R-CHOP treatment, both the peripheral smear and CBC returned to normal limits, leading the authors to conclude that the cold-type AIHA of the patient was attributable to lymphoma. It can thus be hypothesized that CAS was the earliest manifestation of the lymphoma in the patient described herein.

It may be suspected that chronic HBV infection was a risk factor for the patient developing non-Hodgkin lymphoma (NHL). There is an established link between chronic HBV infection and NHL. Furthermore, the risk is higher in Asian populations, such as in Turkey (12).

In conclusion, it is imperative to investigate the underlying causes in patients newly diagnosed with CAD. Cold AIHA may serve as an early indicator of lymphoma that can manifest years prior, necessitating routine monitoring for these patients. In the case that hemoglobin levels are stable in the CBC, and significant fluctuations are observed in the erythrocyte count, hematocrit, mean corpuscular volume and mean corpuscular hemoglobin concentration, particularly if they do not match the clinical presentation of the patient, then CAD should be considered.

## Figures and Tables

**Table I:** Complete blood count of unheated and heated blood samples.

Test/parameter	Unheated sample	Heated sample	Reference range	Units
WBC	6.6	6.2	4-10.3	10³/µl
Neutrophils	5.7	4.6	2.1-6.1	10³/µl
Lymphocytes	0.4	1	1.3-3.5	10³/µl
Monocytes	0.5	0.5	0.3-0.9	10³/µl
Eosinophils	0	0.1	0-0.5	10³/µl
Basophils	0	0	0-0.2	10³/µl
RBC	1.72	4.73	4-5.7	10^6^/µl
Hemoglobin	14.2	14.5	12-16	g/dl
Hematocrit	19.1	41.9	36-46	%
MCV	110.8	88.6	80.7-95.5	fl
MCH	82.7	30.7	27.2-33.5	pg
MCHC	74.6	34.7	32.7-35.6	g/dl
RDW	14.5	13.6	11.8-14.3	%
Platelets	192	289	156-373	10³/µl
MPV	9.9	8.4	6.9-10.8	fl
Plateletcrit	0.189	0.249	0.20-0.36	%

WBC, white blood cell; RBC, red blood cell; MCV, mean corpuscular volume; MCH, mean corpuscular hemoglobin; MCHC, mean corpuscular hemoglobin concentration; RDW, red cell distribution width; MPV, mean platelet volume.

## Data Availability

The datasets used and/or analyzed during the current study are available from the corresponding author on reasonable request.

## References

[1] Gabbard AP, Booth GS (2020). Cold agglutinin disease. Clin Hematol Int.

[2] Berentsen S (2016). Cold agglutinin disease. Hematology Am Soc Hematol Educ Program.

[3] Jäger U, Barcellini W, Broome CM, Gertz MA, Hill A, Hill QA, Jilma B, Kuter DJ, Michel M, Montillo M (2020). Diagnosis and treatment of autoimmune hemolytic anemia in adults: Recommendations from the First International Consensus Meeting. Blood Rev.

[4] Röth A, Barcellini W, D'Sa S, Miyakawa Y, Broome CM, Michel M, Kuter DJ, Jilma B, Tvedt THA, Fruebis J (2021). Sutimlimab in cold agglutinin disease. N Engl J Med.

[5] Pham HP, Wilson A, Adeyemi A, Miles G, Kuang K, Carita P, Joly F (2022). An observational analysis of disease burden in patients with cold agglutinin disease: Results from a large US electronic health record database. J Manag Care Spec Pharm.

[6] Terán Brage E, Fonseca Santos M, Lozano Mejorada R, García Domínguez R, Olivares Hernández A, Amores Martín A, Vidal Tocino R, Fonseca Sánchez E (2022). Autoimmune haemolytic anaemia due to cold antibodies in a renal cancer patient. Case Rep Oncol.

[7] Taberner K, House AA, Haig A, Hsia CC (2023). A case of renal iron overload associated with cold agglutinin disease successfully managed by rituximab. Clin Hematol Int.

[8] Portich JP, Blos B, Sekine L, Franz JPM (2023). Cold agglutinin syndrome secondary to splenic marginal zone lymphoma: A case report. Hematol Transfus Cell Ther.

[9] Lin H, Feng D, Tao S, Wu J, Shen Y, Wang W (2023). A patient with the highly suspected B cell lymphoma accompanied by the erythrocytes cold agglutination: Case report. Medicine (Baltimore).

[10] Goyal K, Sharma R, Garg S (2020). Intestinal diffuse large B-cell lymphoma preceding cold agglutinin disease. J Appl Hematol.

[11] Ranjan V, Dhingra G, Gupta N, Khillan K, Rana R (2022). Cold agglutinin autoimmune haemolytic anaemia as an initial presentation of diffuse large B cell lymphoma: A case study. Curr Med Res Pract.

[12] Gentile G, Arcaini L, Antonelli G, Martelli M (2023). Editorial: HBV and lymphoma. Front Oncol.

